# Hydroxychloroquine administration exacerbates acute kidney injury complicated by lupus nephritis

**DOI:** 10.1186/s13075-021-02700-x

**Published:** 2022-01-03

**Authors:** Ning An, Chen Yang, Hong-Luan Wu, Yun Guo, Xi-Jie Huang, Tong-Sheng Huang, Zhi-Hong Wu, Jing Xue, Rui-Hong Chen, Zhi-Hang Li, Qing-Jun Pan, Hua-Feng Liu

**Affiliations:** Key Laboratory of Prevention and Management of Chronic Kidney Disease of Zhanjiang City, Institute of Nephrology, Affiliated Hospital of Guangdong Medical University, 57 Renmin Road, Zhanjiang, 524001 Guangdong China

**Keywords:** Hydroxychloroquine, Lupus nephritis, Proteinuria, Tubular epithelial cells, Acute kidney injury, Repair

## Abstract

**Background:**

Hydroxychloroquine (HCQ) has been recommended as a basic treatment for lupus nephritis (LN) during this decade based on its ability to improve LN-related renal immune-mediated inflammatory lesions. As a classical lysosomal inhibitor, HCQ may inhibit lysosomal degradation and disrupt protective autophagy in proximal tubular epithelial cells (PTECs). Therefore, the final renal effects of HCQ on LN need to be clarified.

**Method:**

HCQ was administered on spontaneous female MRL/lpr LN mice with severe proteinuria daily for 4 weeks. Moreover, the MRL/lpr mice with proteinuric LN were subjected to cisplatin-induced or unilateral ischemia/reperfusion (I/R)-induced acute kidney injury (AKI) after 2 weeks of HCQ preadministration.

**Results:**

As expected, HCQ treatment increased the survival ratio and downregulated the levels of serum creatinine in the mice with LN, ameliorated renal lesions, and inhibited renal interstitial inflammation. Unexpectedly, HCQ preadministration significantly increased susceptibility to and delayed the recovery of AKI complicated by LN, as demonstrated by an increase in PTEC apoptosis and expression of the tubular injury marker KIM-1 as well as the retardation of PTEC replenishment. HCQ preadministration suppressed the proliferation of PTECs by arresting cells in G1/S phase and upregulated the expression of cell cycle inhibitors. Furthermore, HCQ preadministration disrupted the PTEC autophagy-lysosomal pathway and accelerated PTEC senescence.

**Conclusion:**

HCQ treatment may increase susceptibility and delay the recovery of AKI complicated by LN despite its ability to improve LN-related renal immune-mediated inflammatory lesions. The probable mechanism involves accelerated apoptosis and inhibited proliferation of PTECs via autophagy-lysosomal pathway disruption and senescence promotion.

## Background

Lupus nephritis (LN) is a form of kidney inflammation that is a common and severe complication of systemic lupus erythematosus (SLE) and is characterized by proteinuria, hematuria, and even renal function damage [1, 2]. LN can lead to end-stage renal disease (ESRD) and is the major cause of death in SLE [3]. Over the past decade, growing clinical evidence has demonstrated that antimalarial drugs, mainly hydroxychloroquine (HCQ), show outstanding effectiveness and safety in the treatment of LN [4]. Several authoritative guidelines, such as KDIGO [5, 6], and EULAR/ACR [7], recommend HCQ, in addition to corticosteroids and immunosuppressants, as a basic treatment for LN. Mechanistically, HCQ accumulates in lysosomes, elevating their pH values and resulting in impaired lysosomal proteolysis, which suppresses autoantigen presentation, inhibits endosomal Toll-like receptor signaling, and decreases proinflammatory cytokine production, to suppress immune cell-mediated inflammatory responses [8, 9].

Besides the immune system, HCQ accumulates in other organs, especially those rich in lysosomes, such as the kidneys. HCQ concentration in these organs is dozens of times that in the blood [10]. Renal proximal tubular epithelial cells (PTECs), which are mainly involved in reabsorption of filtered proteins and other glomerular filtrates, contain high levels of lysosomes. Under conditions involving LN-related massive proteinuria, PTECs display protein overloading due to abundant urinary protein reabsorption, which enhances the degradative burden of PTEC lysosomes. In addition, autophagy induces an adaptive response in PTECs under stress conditions to maintain cellular homeostasis, while lysosomes act as the key terminus of the autophagy pathway [11].

Our previous studies demonstrated that the activation of the autophagy-lysosomal pathway was essential in the response of PTECs to urinary proteins [12, 13], and inhibition of the autophagy-lysosomal pathway by chloroquine (CQ) promoted PTEC injury by urinary proteins [12]. As mentioned above, severe proteinuria is a common clinical manifestation of LN, and excessive inhibition of lysosomes may exacerbate PTEC injury in patients with LN with severe proteinuria. Moreover, patients with LN are prone to acute kidney injury (AKI), which is an important risk factor for poor renal prognosis and death [14]. In the last decade, many studies have demonstrated that autophagy activation in PTECs protects against AKI, and inhibition of the autophagy-lysosomal pathway by CQ promotes AKI [15].

Thus, we inferred that HCQ may act as a double-edged sword in LN with massive proteinuria: HCQ may relieve renal immune inflammatory injuries by inhibiting immune cell lysosomes but may also exacerbate renal injuries by inhibiting lysosomal degradation and disrupting protective autophagy in PTECs. Thus, clarification of the final renal effects of HCQ on LN is needed.

## Methods

### Reagents and antibodies

DMEM (C11995500BT), fetal bovine serum (10270106), and penicillin-streptomycin (15140122) were obtained from Gibco (New York, NY, USA). HCQ sulfate (90527) and 5-bromo-2′-deoxyuridine (BrdU) were obtained from Sigma-Aldrich (Louis, MO USA). Antibodies against p62/sequestosome 1 (ab56416), CD3 (ab16669), p53 (ab31333), BrdU (ab2284), collagen I (ab34710), and α-smooth muscle actin (α-SMA, ab124964) were purchased from Abcam (Cambridge, MA, USA). Antibodies against phospho-histone H3 (9701s) and p21 (2947S) were obtained from Cell Signaling. Antibodies against microtubule-associated protein 1 light chain 3B (LC3, L7543) were purchased from Sigma-Aldrich. Anti-HAVCR1/TIM1/KIM-1 (kidney injury molecule-1, KIM-1, AF1817) was obtained from R&D Systems (Minneapolis, MN, USA). β-actin antibody (sc-47778) was purchased from Santa Cruz (Dallas, TX, USA). Rat anti-mouse F4/80 antibody (MCA497GA) and Clarity™ Western ECL Substrate (170-5060) were purchased from Bio-Rad (Hercules, CA, USA). Alexa 594-conjugated donkey anti-rat IgG (A-21209) was obtained from Thermo Fisher Scientific (Eugene, OR, USA). Horseradish peroxidase (HRP)-conjugated secondary antibodies (A0216, A0208, A02181), RIPA lysis buffer (P0013E), Cell Counting Kit-8 (CCK-8, C0037), propidium iodide (PI, ST511), and phenylmethanesulfonyl fluoride (PMSF, ST506) were purchased from Beyotime (Shanghai, China). A BCA Protein Assay Kit (23225) was purchased from Pierce (Rockland, IL, USA). A phosphatase inhibitor cocktail (P1260) was purchased from Applygen (Beijing, China).

### Animal experiments

Female MRL/MpJ-Faslpr/J (MRL/lpr) mice were obtained from the Shanghai SLAC Laboratory Animal Co., Ltd. (Shanghai, China, 000485). Mice were housed at the Animal Center of Guangdong Medical University. All procedures of this study were approved by the Animal Experimentation Ethics Committee of Guangdong Medical University (NO. GDY1602005).

MRL/lpr mice developed proteinuria above 0.3 mg/ml in spot urine was considered as mice with LN. The concentration of urinary protein above 1 mg/ml was considered as severe proteinuria in the present study [16]. Mice (12-week old) with LN and severe proteinuria (concentration above 1 mg/ml) were randomly received HCQ (HCQ group) or saline (CON group). According to the dose conversion between mice and humans and for quick achievement of steady-state blood concentrations, mice with proteinuric LN were orally administered HCQ (200 mg/kg body weight) daily over 4 weeks. The control LN mice were orally administrated with the same volume of saline during the same time.

In the cisplatin-induced AKI study, mice with severe LN were treated with HCQ (80 mg/kg body weight) daily for 2 weeks and then administered a single intraperitoneal injection of cisplatin (15 mg/kg) to induce AKI. HCQ was continuously administered after cisplatin injection. The control LN mice with AKI were gavage administrated with the same volume of saline during the same time. Mice were anesthetized by intraperitoneal injection of an overdose of sodium pentobarbital (100 mg/kg) and were sacrificed at 96 h after AKI induction.

In the unilateral ischemia-reperfusion (I/R)-induced AKI study, mice were administered HCQ daily for 2 weeks and then subjected to unilateral I/R injury by clamping the right kidney pedicle for 45 min using a surgical approach under pentobarbital sodium (50 mg/kg)-induced anesthesia. The left kidney was left untouched. The control LN mice with AKI were orally administrated with the same volume of saline. Then, the mice were treated with saline or HCQ in succession and euthanized on day 3, day 7, or day 14.

### Renal function assessment

Mouse urinary protein was quantified using the Quick Start Bradford Protein Assay (Bio-Rad, 1-800-424-6723), and serum creatinine (Scr) was detected by a creatinine assay kit (Nanjing Jiancheng Bioengineering Institute, C011-2-1) according to the manufacturer’s instructions.

### Histopathology

Renal tissue sections (3 μm) were stained using the periodic acid-Schiff (PAS) method, Masson’s trichrome staining, or Sirius red staining according to standard procedures [16]. Examination and scoring of sections were performed by two professional pathologists in a blinded manner. Glomerular injury was scored from 0 to 3 according to the pervious study [17]. Tubular injury was scored from 0 to 4 as described previously [18].

Senescence-associated-β-galactosidase (SA-β-gal) staining was performed as described previously [16]. The positive areas (%) for tubular staining were measured using ImageJ software (NIH, USA).

### Immunochemistry and immunofluorescence

Immunochemistry was performed as previously described [19]. The number of positive cells in ten × 400 magnification fields per sample was counted (Olympus, BX64 and DP74, Tokyo, Japan). The positive areas (%) for target signals were measured using ImageJ software (NIH, USA).

Immunofluorescence was performed as previously described [19]. Ten fields per sample were collected at × 400 magnification using a TCS SP5 II confocal microscope (Leica Microsystems, Germany). Positive cell staining was further semiquantified as described in the immunochemistry section.

### TUNEL assay

TUNEL staining was performed by utilizing a DeadEnd™ Fluorometric TUNEL System (Promega, G3250). Pictures of positive cells were captured and analyzed as mentioned previously in the immunofluorescence study [12].

### Real-time PCR

As previously described [19], real-time PCR was performed on a LightCycler® 480 System (Roche, USA) using the TB Green PCR Kit (TaKaRa, RR820A). The primers used in this study included primers for mouse TNF-α and IL-1β and glyceraldehyde-3-phosphate dehydrogenase (GAPDH). The primer sequences were as follows: mouse TNF-α forward: 5′-CATGAGCACAGAAAGCATGATCCG-3′ and reverse: 5′-AAGCAGGAATGAGAAGAGGCTGAG-3′; mouse IL-1β forward: 5′-CTTCAGGCAGGCAGTATCACTCAT-3′ and reverse: 5′-TCTAATGGGAACGTCACACACCAG-3′; mouse GAPDH forward: 5′-GCATGGCCTTCCGTGTTC-3′ and reverse: 5′-GATGTCATCATACTTGGCAGGTTT-3′. The level of the mRNA of interest was normalized to the GAPDH mRNA level.

### Western blot analysis

Protein expression was analyzed by Western blot analysis as previously described [19]. The signals were detected by an Azure C500 Western Blot Imaging System and then quantified by using ImageJ software (NIH).

### Purification of urinary proteins

Urinary proteins were extracted from the urine of patients with biopsy-proven and untreated severe LN using an ammonium sulfate precipitation method as described previously [12].

### Cell culture and treatment

Human kidney tubular epithelial cells (HK-2) were obtained from the American Type Culture Collection (ATCC, CRL-2190TM) and were as cultured as described previously [19]. Urinary protein (10 mg/ml) was used to stimulate HK-2 cells in vitro to mimic proteinuria-induced tubular injury in severe LN as described previously [12]. Two to 10 μg/ml HCQ was used to stimulate HK-2 cells according to the pharmacokinetics of HCQ during long-term use [20].

### Cell proliferation analysis

CCK-8 assays were used for the detection of cell proliferation according to the manufacturer’s instructions. The absorbance at 450 nm was measured by a microplate reader (BioTek, ELx800, Winooski, VT, USA).

### Flow cytometric analysis

Cell cycle analysis was performed by PI DNA staining and subsequent flow cytometric analysis with the FACSCanto II platform (BD, FACSCanto II, USA). For detection of lysosomal degradation, the mean fluorescence intensity of green degradable compounds from DQ-ovalbumin (Invitrogen) in HK-2 cells was analyzed by flow cytometry as described previously [19]. For detection of cell proliferation, the mean fluorescence intensity of CFSE (C0051, Beyotime) in the HK-2 cells was analyzed by flow cytometry.

### Statistical analysis

Data are presented as the mean ± standard error of the mean (SEM). For comparisons between two groups, Student’s *t* test was used; for comparisons among multiple groups, one-way analysis of variance (ANOVA) was used followed by Tukey’s post hoc tests. *P*< 0.05 was defined as significant in this study. Data analysis was performed, and graphics were created using GraphPad Prism 5 (GraphPad Software, San Diego, CA, USA).

## Results

### HCQ administration alleviated renal injury in mice with LN

**Fig. 1:**
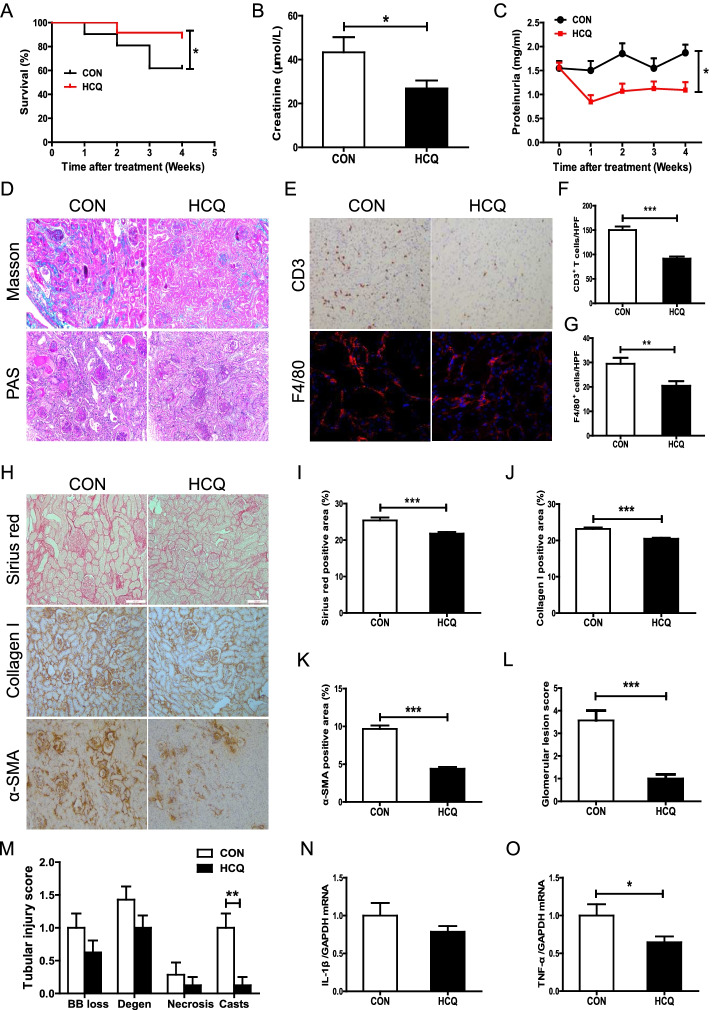
HCQ increases the survival rate and protects against renal injury in mice with proteinuric LN. **A** Survival rate of the mice with proteinuric LN. CON (*n* = 21) and HCQ (*n* = 24). **B** Scr levels in the mice with proteinuric LN administered HCQ for 4 weeks. **C** Urinary protein levels in the mice with LN administered HCQ for 4 weeks. **D** Representative PAS staining and Masson staining images of the renal tissue of the mice with proteinuric LN. **E** Representative immunohistochemical staining of renal CD3 and immunofluorescence staining images of renal F4/80 in the mice with proteinuric LN. **F**, **G** Quantitative analysis of renal CD3- or F4/80-positive cells per high-power field (HPF). **H** Representative Sirius red staining images and immunohistochemical staining images of renal collagen I and α-SMA in the mice with proteinuric LN. **I**–**K** Quantitative analysis of renal Sirius red staining and collagen I- and α-SMA-positive areas per HPF. **L**, **M** Quantitative analysis of glomerular and tubular injury scores of the mice with proteinuric LN. **N** Renal mRNA level of IL-1β in the mice with LN administered HCQ for 4 weeks. **O** Renal mRNA level of TNF-α in the mice with LN administered HCQ for 4 weeks. Each bar represents the mean ± SEM for groups of 14 to 21 mice. *P < 0.05, **P < 0.01, and ***P < 0.001. Original magnification× 200

In clinical use, short-term high-dose HCQ has been used to maintain the therapeutic blood concentration of HCQ in patients that less response to the standard dose. Even 800 mg or 1200 mg per day was used to achieve the therapeutic 750 ng/ml blood level of HCQ [21]. According to the dose conversion between human and mouse, the dose used in mouse is recommended to 12-fold more as that used in human [22, 23]. Thus, mice treated with 200 mg/kg/day (means 1000 mg/kg/day in human) for 1 month to explore the potential therapeutic effect of HCQ on proteinuric LN. After administration of HCQ, as shown in Fig. 1A, we found that the treatment significantly improved the survival rate of the MRL/lpr mice with proteinuria compared with that of the saline-treated controls (CONs). The concentration of urinary protein in the control MRL/lpr mice increased continually in an age-dependent manner, while treatment with HCQ notably suppressed the excretion of urinary protein (Fig. 1C). HCQ administration also substantially decreased the concentration of Scr in the mice with LN (Fig. 1B). Renal glomerular lesions, including glomerular cell proliferation, Bowman’s capsule synechiae, necrosis, glomerular segmental sclerosis, and presence of crescents were significantly alleviated in the HCQ-treated mice compared with the CONs (Fig. 1D, L). Renal tubulointerstitial damage, such as PTEC vacuole degeneration and transdifferentiation (tubular α-SMA expression), tubular atrophy, and collagen I deposition in the renal interstitium, was also attenuated by HCQ administration (Fig. 1D, M, and H–K). Furthermore, HCQ treatment significantly inhibited renal interstitial infiltration of CD3^+^ T cells and F4/80^+^ macrophages (Fig. 1E–G), as well as the renal expression of the proinflammatory cytokines IL-1β and TNF-α (Fig. 1N, O). The results indicated that HCQ treatment could improve the damage to the renal structure and function in the mice with massive proteinuric LN.

### HCQ administration increased susceptibility to AKI in mice with LN

**Fig. 2:**
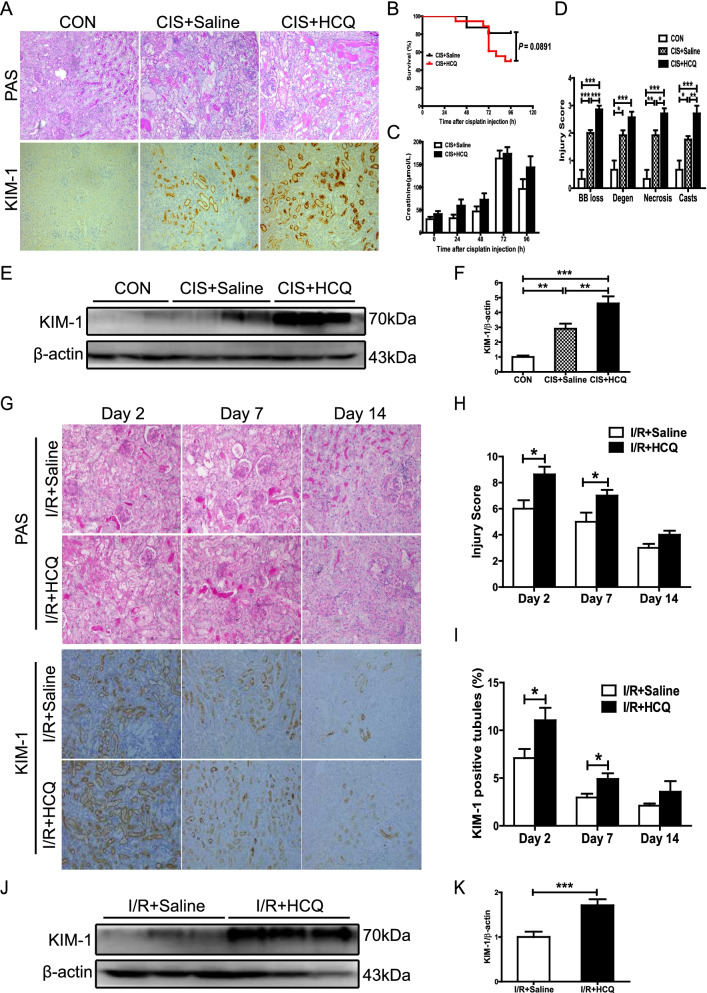
HCQ exacerbates renal injury in the mice with proteinuric LN subjected to AKI. **A** Representative PAS staining images and immunohistochemical staining of renal KIM-1 in the mice with proteinuric LN subjected to cisplatin-induced AKI. **B** Survival rate of the mice with proteinuric LN subjected to cisplatin-induced AKI. CIS + saline (*n* = 16) and CIS + HCQ (*n* = 18). **C** Scr levels in the mice with proteinuric LN subjected to cisplatin-induced AKI. **D** Quantitative analysis of renal injury scores of the mice with proteinuric LN subjected to cisplatin-induced AKI. **E** Western blotting of renal KIM-1 in the mice with proteinuric LN subjected to cisplatin-induced AKI. **F** Quantitative analysis of KIM-1 expression in the mice with proteinuric LN subjected to cisplatin-induced AKI by Western blotting. **G** Representative PAS staining images and immunohistochemical staining of renal KIM-1 in the mice with proteinuric LN subjected to I/R-induced AKI. **H** Quantitative analysis of the renal injury scores of the mice with proteinuric LN subjected to I/R-induced AKI. **I** Quantitative analysis of renal KIM-1 in the mice with proteinuric LN subjected to I/R-induced AKI. **J** Western blotting analysis of renal KIM-1 in the mice with proteinuric LN subjected to I/R-induced AKI. **K** Quantitative analysis of KIM-1 expression in the mice with proteinuric LN subjected to I/R-induced AKI by Western blotting. Each bar represents the mean ± SEM. For the cisplatin-induced AKI experiments, there were more than 7 mice in each group; for the I/R-induced AKI experiments, there were more than 5 mice in each group at each time point. *P < 0.05, **P < 0.01, and ***P < 0.001. Original magnification × 200

According to the 2019 recommendations for the management of LN, the dose of HCQ is recommended not more than 5 mg/kg/day in clinical use to avoid potential toxicity [7]. However, 6.5 mg/kg/day have been proved to be therapeutic efficacy but not 5 mg/kg/day. And one tablet of HCQ contains 200 mg, and most patients with LN take two tablets a day in clinical applications, which closer to 6.5 mg/kg/day. According to the dose conversion between human and mouse, the dose used in mouse is recommended to 12-fold more as that used in human [22, 23]. Thus, we used 80 mg/kg/day in LN mice subjected to AKI. In contrast to the efficacy of HCQ in the mice with proteinuric LN, preadministration of HCQ increased the susceptibility of the mice with proteinuric LN to developing AKI, regardless of whether they were treated with nephrotoxic drugs or were subjected to unilateral I/R injury. As shown in Fig. 2B and C, in the cisplatin-induced AKI models, an increase in the Scr levels and a slight decrease in the survival rate were found in the mice with LN and HCQ preadministration compared with the saline-treated mice. HCQ preadministration also notably aggravated PTEC brush border loss and vacuolar degeneration, renal tubular necrosis, and cast formation in the cisplatin-induced AKI models (Fig. 2A, D). Furthermore, the expression of KIM-1 was significantly increased in the HCQ-pretreated mice with cisplatin-induced AKI (Fig. 2A, E, F). An increase in AKI development was also found in the mice with proteinuric LN suffering from unilateral I/R injury, as shown by the notably aggravated renal pathological lesions and elevated expression of KIM-1 (Fig. 2G–K).

**Fig. 3:**
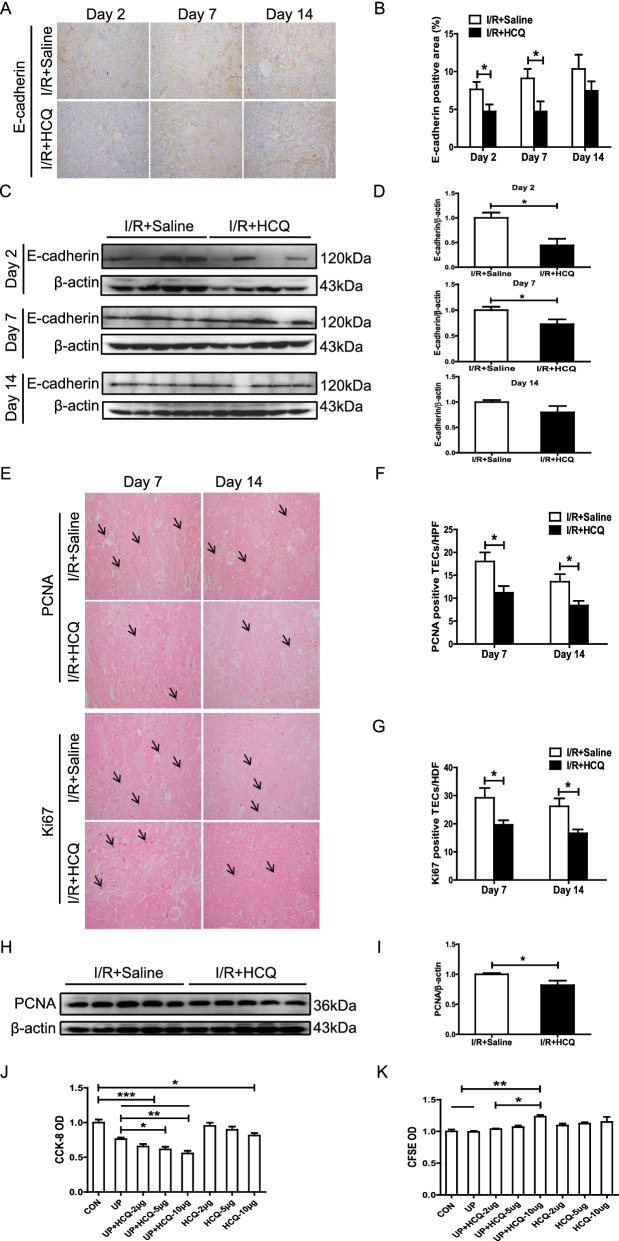
HCQ delays renal repair in the mice with proteinuric LN after AKI. **A** Representative immunohistochemical staining of renal E-cadherin in the mice with proteinuric LN subjected to I/R-induced AKI. **B** Quantitative analysis of renal E-cadherin in the mice with proteinuric LN. **C** Western blotting of renal E-cadherin in the mice with proteinuric LN. **D** Quantitative analysis of renal E-cadherin expression in the mice with proteinuric LN subjected to I/R-induced AKI by Western blotting. **E** Representative immunohistochemical staining and renal PCNA and Ki67 staining of the mice with proteinuric LN subjected to I/R-induced AKI. **F**, **G** Quantitative analysis of renal PCNA and Ki67 in the mice with proteinuric LN subjected to I/R-induced AKI. **H** Western blotting analysis of renal PCNA in the mice with proteinuric LN. **I** Quantitative analysis of renal PCNA expression in the mice with proteinuric LN by Western blotting. **J** CCK-8 analysis of the proliferation of HK-2 cells. **K** CFSE staining and flow cytometric analysis of HK-2 cells. Each bar represents the mean ± SEM, with more than 5 mice in each group at each time point. *P < 0.05, **P < 0.01, and ***P < 0.001. Original magnification × 200

Kidney repair after AKI is characterized by restoration of the polarity of PTECs. We detected the expression of the epithelial cell marker E-cadherin, a major component of tubular adherent proteins that maintains intercellular contacts and PTEC polarity in renal tissues. As shown in Fig. 3A–D, HCQ preadministration suppressed the restoration of tubular E-cadherin expression when the mice with proteinuric LN suffered from unilateral I/R injury on day 2 and day 7. Next, we found that the rate of PCNA-positive and Ki67-positive PTECs was decreased in the HCQ-pretreated mice with LN compared with the control mice with LN on day 7 and day 14 after renal unilateral I/R injury (Fig. 3E–G). The downregulation of renal PCNA expression was further confirmed by Western blotting in the HCQ-pretreated mice with LN (Fig. 3H, I). In the in vitro study, HCQ also suppressed the proliferation of HK-2 cells stimulated by urinary proteins from patients with LN in a dose-dependent manner (Fig. 3J, K).

### HCQ administration promoted PTEC apoptosis and arrested cell cycle progression at G1/S phase in LN

**Fig. 4:**
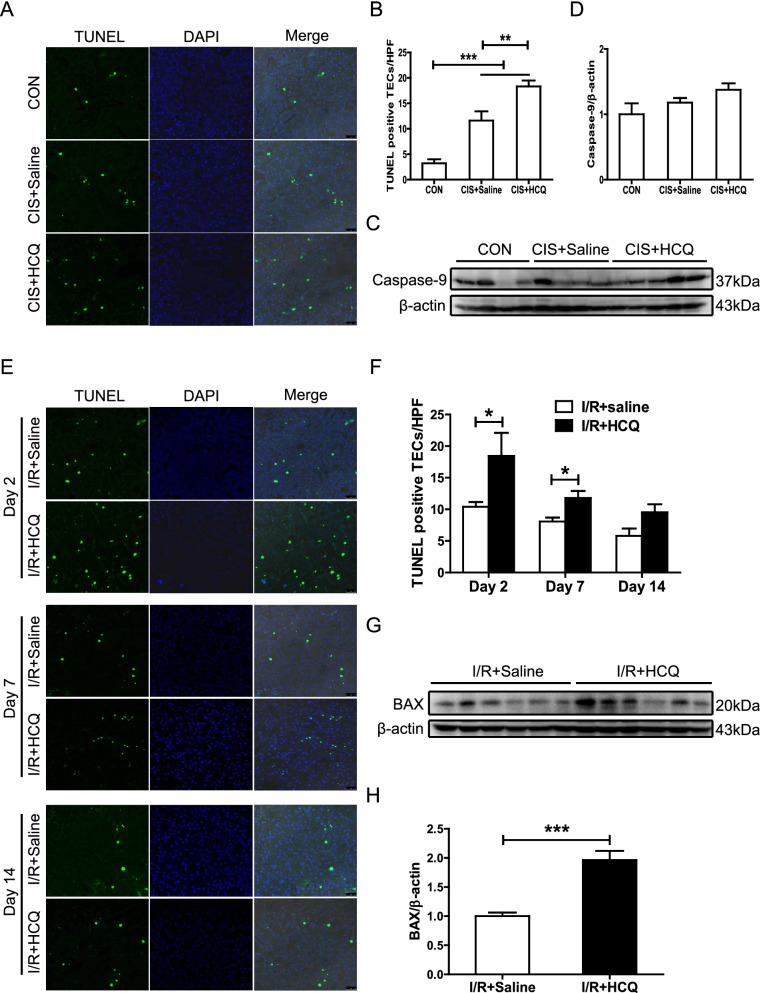
HCQ promotes PTEC apoptosis in the mice with proteinuric LN subjected to AKI. **A** Representative renal TUNEL staining images of the mice with proteinuric LN subjected to cisplatin-induced AKI. **B** Quantitative analysis of renal TUNEL staining of the mice with proteinuric LN subjected to cisplatin-induced AKI. **C** Western blotting of renal caspase-9 expression in the mice with proteinuric LN subjected to cisplatin-induced AKI. **D** Quantitative analysis of renal caspase-9 expression in the mice with proteinuric LN subjected to cisplatin-induced AKI by Western blotting. **E** Representative renal TUNEL staining images of the mice with proteinuric LN subjected to I/R-induced AKI. **F** Quantitative analysis of renal TUNEL staining in the mice with proteinuric LN subjected to I/R-induced AKI. **G** Western blotting of renal BAX expression in the mice with proteinuric LN subjected to I/R-induced AKI. **H** Quantitative analysis of renal BAX expression in the mice with proteinuric LN subjected to I/R-induced AKI by Western blotting. Each bar represents the mean ± SEM. In the cisplatin-induced AKI experiments, there were more than 7 mice in each group; in the I/R-induced AKI experiments, there were more than 5 mice in each group at each time point. *P < 0.05, **P < 0.01, and ***P < 0.001. Original magnification × 200

PTEC apoptosis plays a critical role in the development of AKI. We found that HCQ preadministration significantly exacerbated cisplatin-induced PTEC apoptosis (Fig. 4A, B). Similar results were also found in the mice with proteinuric LN subjected to unilateral I/R (Fig. 4E, F). Synchronously, HCQ preadministration also significantly increased the expression of the apoptosis-related proteins caspase-9 and BAX in the renal tubules of the mice with proteinuric LN and AKI (Fig. 4C, D, G, H).

**Fig. 5:**
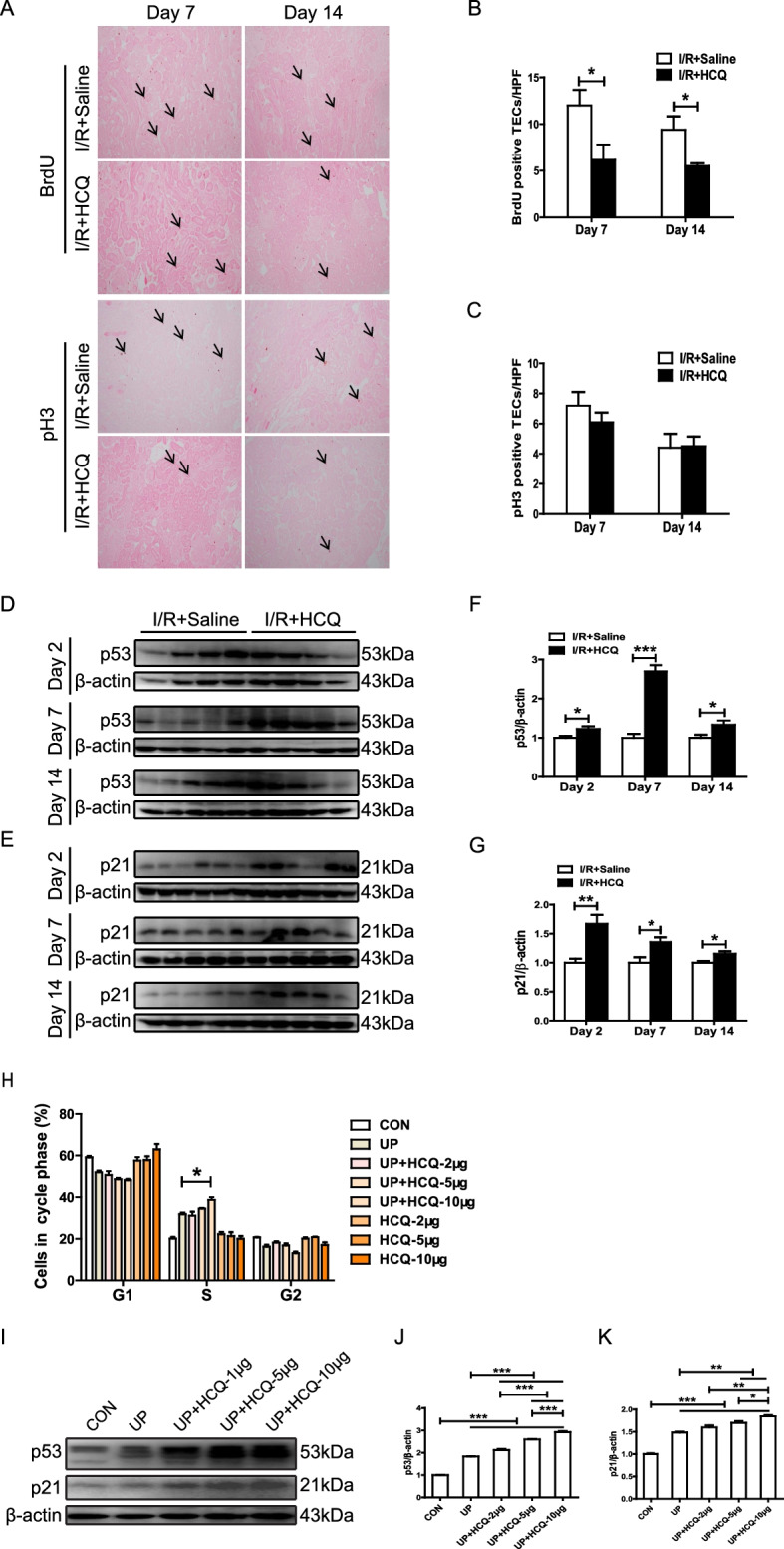
HCQ promotes PTEC G1/S arrest in the mice with proteinuric LN. **A** Representative immunohistochemical staining and quantitative analysis of renal BrdU and Ki67 in the mice with proteinuric LN. **B**, **C** Quantitative analysis of renal BrdU and Ki67 in the mice with proteinuric LN. **D**–**G** Western blotting and quantitative analysis of renal p53 and p21 in the mice with proteinuric LN subjected to I/R-induced AKI. **H** PI staining and flow cytometric analysis of HK-2 cells. **I**–**K** Western blotting and quantitative analysis of p53 and p21 expression in the HK-2 cells after exposure to HCQ and urinary proteins extracted from patients with LN. Each bar represents the mean ± SEM of at least 3 independent experiments, with more than 5 mice in each group at each time point. *P < 0.05, **P < 0.01, and ***P < 0.001. Original magnification × 200

We next investigated whether HCQ preadministration suppressed PTEC proliferation via arresting cell cycle progression. HCQ treatment notably inhibited the uptake of BrdU by PTECs in the mice with proteinuric LN after cisplatin- or I/R-induced AKI (Fig. 5A, B). However, the proportion of pH 3-positive PTECs, an indicator of mitosis and cell cycle arrest during the G2/M phase, was not affected by HCQ preadministration (Fig. 5A, C). In an in vitro study, the ratio of HK-2 cells arrested in S phase after treatment with urinary proteins from patients with LN was also significantly increased by HCQ stimulation (Fig. 5H). Mechanistically, the renal expression of the cell cycle inhibitors p53 and p21 was notably elevated in the mice with LN that were pretreated with HCQ and subjected to unilateral I/R-induced AKI (Fig. 5D–G). Moreover, in accordance with the in vivo study, HCQ treatment dose-dependently upregulated the expression of p53 and p21 in HK-2 cells treated with urinary proteins from LN samples (Fig. 5I–K).

### HCQ administration accelerated cellular senescence and impaired the autophagy-lysosomal pathway in PTECs in LN

**Fig. 6:**
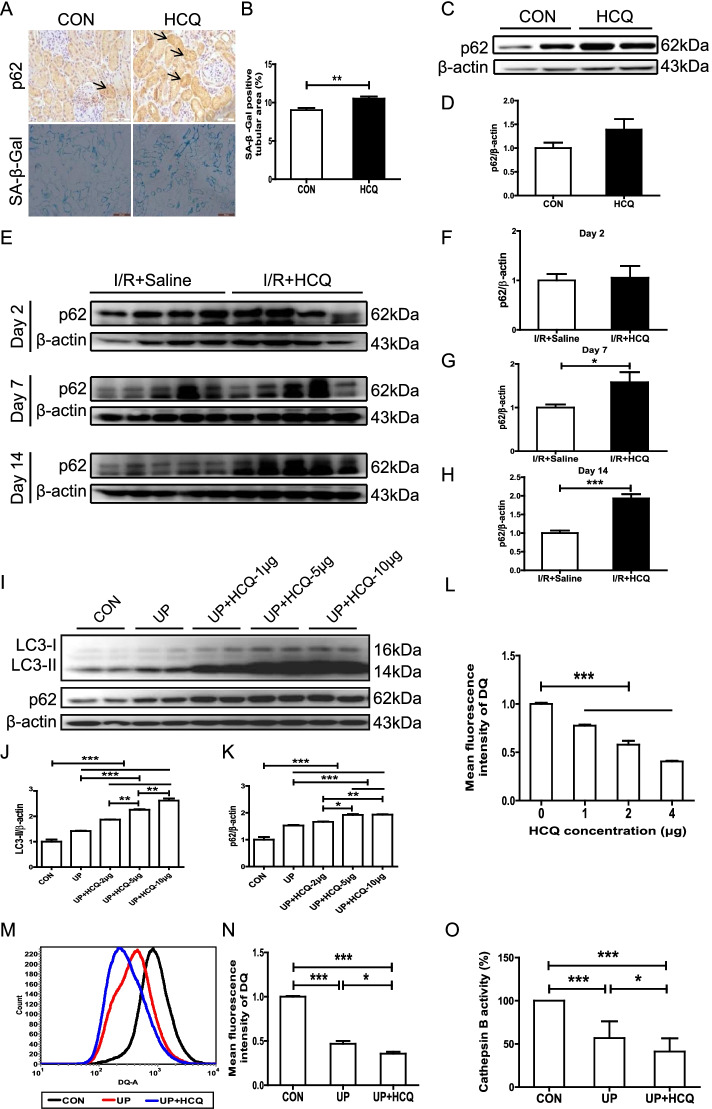
HCQ impairs the autophagy-lysosomal pathway and accelerates senescence in PTECs. **A** Representative immunohistochemical staining of p62 and SA-β-gal in the mice with proteinuric LN. **B** Quantitative analysis of the SA-β-gal-positive tubular area. **C**, **D** Western blotting analysis and quantitative analysis of renal p62 in the mice with proteinuric LN. **E**–**H** Western blotting analysis and quantitative analysis of renal p62 in the mice with proteinuric LN subjected to unilateral I/R-induced AKI. **I**–**K** Western blotting analysis and quantitative analysis of LC3 and p62 in HK-2 cells. **L** Flow cytometric analysis of DQ-ovalbumin staining in HK-2 cells after exposure to HCQ. **J**, **K** Flow cytometric analysis of DQ-ovalbumin staining in HK-2 cells after exposure to urinary proteins from patients with LN and HCQ. **L** Detection of cathepsin B activity in HK-2 cells after exposure to urinary proteins from patients with LN and HCQ. Each bar represents the mean ± SEM of at least 3 independent experiments, with more than 5 mice in each group at each time point. *P < 0.05, **P < 0.01, and ***P < 0.001

Accelerated cellular senescence in tubular cells exacerbates injury and incomplete repair due to AKI. As shown in Fig. 6A, HCQ administration significantly elevated the staining of SA-β-gal in PTECs. As impairment of lysosome-dependent autophagy is tightly associated with cellular senescence, how HCQ affects the autophagy-lysosomal pathway of PTECs under LN conditions was also investigated. As shown in Fig. 6A–D, HCQ treatment impaired the renal autophagy-lysosomal pathway in the mice with proteinuric LN and induced PTEC senescence, as demonstrated by the accumulation of the autophagy-related protein p62. Similarly, renal p62 accumulation was also detected in the mice with LN pretreated with HCQ that were subjected to I/R-induced AKI (Fig. 6E–H). To further confirm the inhibitory effect of HCQ on the autophagy-lysosomal pathway, we incubated HK-2 cells with urinary proteins extracted from patients with LN and treated them with or without HCQ. The autophagy-lysosomal pathway in the urinary protein-treated HK-2 cells was further impaired by HCQ treatment (Fig. 6I–K). Importantly, we found that HCQ mainly suppressed lysosomal degradation, the terminus of the autophagy-lysosome pathway in PTECs, represented by a decrease in the mean fluorescence intensity of DQ-ovalbumin in a dose-dependent manner (Fig. 6L). In addition, the lysosomal cathepsin B activity was further inhibited by HCQ in the urinary protein-treated HK-2 cells (Fig. 6M–O).

## Discussion

HCQ, combined with standard immunosuppressants, has been proven to retard renal damage occurrence, accelerate renal remission, reduce lupus relapse, and decrease mortality in patients with LN [7, 24]. However, the renoprotective effects of HCQ monotherapy in LN have not been fully elucidated. In the present study, we confirmed for the first time that HCQ monotherapy could also preserve renal function and elevate the survival of LN using spontaneous LN models. In addition to further supporting previous clinical findings, our study provided more direct pathological evidence that HCQ treatment attenuates glomerular and tubulointerstitial lesions in LN [25]. Excessive systemic and kidney immunoinflammatory responses mediate lupus-related renal lesions in LN. Our present data showed that HCQ treatment significantly inhibited renal inflammatory responses. Moreover, we recently reported that HCQ lowered circulating inflammatory cytokines and autoantibodies by rebalancing the immune response of Th17/Treg cells [26]. Thus, HCQ may improve renal lesions and function in LN by suppressing immune cell-mediated systemic and kidney inflammatory responses.

A growing body of evidence has demonstrated that AKI can trigger the development of or aggravate the progression of chronic kidney disease (CKD) [27]. Patients with LN, a classical type of CKD, are prone to AKI [14]. Severe AKI and incomplete recovery from AKI may accelerate the progression of renal lesions in LN [14]. Ischemic and nephrotoxic AKI are two common and major types of experimental models of AKI that mimic the pathophysiology of intrinsic AKI [28, 29]. Ischemic AKI is the serious cause of AKI in both native kidneys and renal allografts. Cisplatin, a common chemotherapeutic agent, can induce AKI in rodent with many pathophysiologic features similar to patients. Although nearly 20% patients with renal biopsy-proven LN developed AKI, the cause of AKI was not well explored [14]. Many experts believe that AKI in glomerulonephritis can result from acute tubular necrosis (ATN) from renal hypoperfusion or drug- or radiocontrast agent-induced tubular epithelial cell injury besides of glomerular conditions [30]. Thus, ischemic- and cisplatin-induced AKI are well accepted models mimicking AKI developed in LN patients. In the present study, when subjected to nephrotoxic drugs or I/R, the mice with proteinuric LN that received long-term preadministration of HCQ developed more severe AKI than those without HCQ preadministration. PTEC apoptosis is a driving pathophysiological mechanism in AKI [31]. We found that HCQ preadministration elevated the rates of PTEC apoptosis associated with an increase in proapoptotic BAX and caspase-9 expression, which may promote PTEC injury induced by cisplatin and I/R in the mice with LN. These results were consistent with our previous findings that CQ aggravated PTEC apoptosis caused by urinary proteins in a mouse model of proteinuric kidney disease [12].

In addition, upon AKI, the remnant PTECs undergo dedifferentiation, proliferation, and redifferentiation to replenish apoptotic and necrotic PTECs [32], which is critical for tubular regeneration and full recovery from AKI [33]. In the present study, we found that the proliferative ability of PTECs after AKI was notably inhibited by HCQ preadministration, consistent with the restoration of tubular redifferentiation indicated by a decrease in the expression of E-cadherin. Both G1/S and G2/M cycle arrest has been implicated in maladaptive kidney repair after AKI [34, 35]. We found that HCQ induced G1/S arrest and increased the expression of p53 and p21 in PTECs. Previous studies have demonstrated that p53 and p21 mediate G1/S arrest to inhibit PTEC proliferation in AKI [35, 36]. In brief, long-term HCQ treatment aggravated PTEC apoptosis and suppressed PTEC proliferation by promoting G1/S cycle arrest, resulting in delayed renal recovery from AKI [37]. Thus, our findings demonstrated for the first time that HCQ preadministration increases susceptibility to AKI in proteinuric LN. Unfortunately, a growing body of literature has confirmed that chronic use of HCQ and CQ may induce organ damage [38, 39], including kidney injury [40].

Increasing evidence has shown that autophagy in kidneys serves as a crucial protective mechanism in response to stress. In LN, under the stimulation of the inflammatory response and massive proteinuria, autophagy is activated to maintain PTEC homeostasis. In the present study, we demonstrated that HCQ notably blocked the autophagy pathway by disrupting lysosomal degradation and simultaneously accelerated senescence in PTECs, which in turn may aggravate PTEC injury in LN. However, the potential risk of HCQ administration is decreased by the weakening of the inflammatory response by HCQ. In contrast, with nephrotoxic and ischemic AKI, two major risk factors directly causing PTEC damage, HCQ preadministration led to susceptibility to AKI in individuals with LN. Blocking the autophagy pathway and accelerating senescence in PTECs by HCQ may reduce cellular resistance to stress. Aged kidneys are susceptible to nephrotoxicity and ischemia [41]. Increased tubular expression of BAX and caspase-3/9 indicates enhanced cell injury with aged kidneys due to PTEC apoptosis [42]. Moreover, the expression of renal p53 and p21 was elevated in aged male mice after ischemia-induced AKI, contributing to the renal regenerative capacity [43]. Pathologically, the accumulated senescent cells are a driving force of kidney aging [44]. Similar to our previous study [16], tubular senescent cells accumulated in inflamed kidneys in the mice with LN and were further increased by HCQ treatment. Although the detailed mechanisms are still unclear, cellular senescence is tightly linked to impaired lysosome-dependent autophagy [45]. For example, autophagy protects against oxidative stress-induced senescence by eliminating damaged mitochondria [46]. Therefore, promoting senescence and impairing the autophagy-lysosomal pathway may be an important mechanism by which HCQ increases apoptosis and suppresses proliferation in PTECs when AKI is complicated by LN. Regrettably, due to the lack of tubule-specific agonists of the autophagy-lysosomal pathway, we could not prove that rescue of the autophagy-lysosomal pathway decreases the susceptibility to AKI in the mice with proteinuric LN and HCQ preadministration, which should be performed in the future.

As mentioned above, we found that a long-term therapeutic dose of HCQ for LN may disrupt autophagy and promote senescence in PTECs to increase the susceptibility to AKI. Recently, HCQ or CQ use in COVID-19 patients was also emphasized that these drugs may worsen AKI by inhibiting autophagy [47–49]. A previous study by Tang et al. [50] found that short-term HCQ therapy attenuated I/R-induced AKI, although the autophagy pathway was also inhibited. The dose used in that study was only 10 mg/kg, which was less than the dose of HCQ used in treatment for LN to maintain an effective blood concentration [51]. The risk of HCQ increases with treatment duration [52]. We inferred that different doses and treatment times of HCQ may lead to the different effects on AKI between Tang’s study and ours. In addition, similar to retinopathy induced by HCQ use, acute renal impairment may inhibit renal elimination of HCQ, which in turn elevates the renal level of HCQ and its toxicity [53–55]. Thus, the nephrologist should pay attention to HCQ use in proteinuric LN patients during AKI, and monitoring the blood level of HCQ is suggested.

## Conclusions

**Fig. 7:**
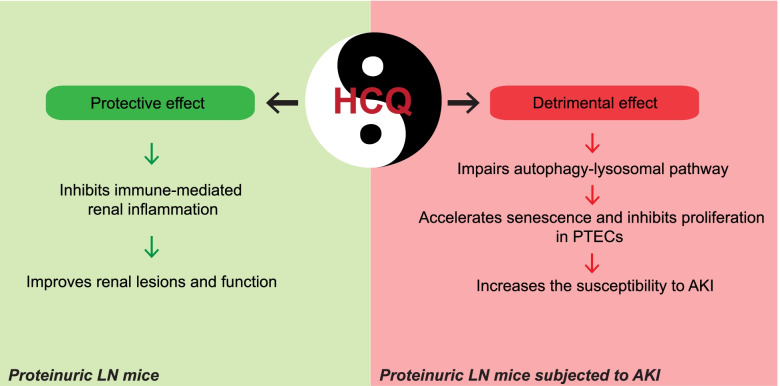
Schematic representation of HCQ as a double-edged sword in the treatment of LN. HCQ may protect the kidneys from lupus-related immunoinflammatory lesions, but long-term HCQ administration for severe proteinuric LN may increase the susceptibility to AKI by disrupting the autophagy-lysosomal pathway and accelerating cellular senescence in PTECs

HCQ treatment would be a double-edged sword in patients with LN with severe proteinuria. On the one hand, HCQ protects the kidneys from lupus-related immunoinflammatory lesions, but on the other hand, long-term HCQ administration in severe proteinuric LN may increase the susceptibility to AKI by disrupting the autophagy-lysosomal pathway and promoting senescence (Fig. 7).

## Data Availability

All data included in this study are available upon request by contact with the corresponding author.

## Abbreviations

ACR: American College of Rheumatology
AKI: Acute kidney injury
CKD: Chronic kidney disease
CQ: Chloroquine
ESRD: End-stage renal disease
EULAR: European League Against Rheumatism
GAPDH: Glyceraldehyde-3-phosphate dehydrogenase
HCQ: Hydroxychloroquine
HRP: Horseradish peroxidase
I/R: Ischemia-reperfusion
KDIGO: Kidney Disease: Improving Global Outcomes
LC3: Microtubule-associated protein 1 light chain 3B
LN: Lupus nephritis
PAS: Periodic acid-Schiff
PTECs: Proximal tubular epithelial cells
SA-β-gal: Senescence-associated-β-galactosidase
Scr: Serum creatinine
SEM: Standard error of the mean
SLE: Systemic lupus erythematosus
α-SMA: α-Smooth muscle actin

## References

[1] Almaani S, Meara A, Rovin BH (2017). Update on lupus nephritis. Clin J Am Soc Nephrol.

[2] Dorner T, Furie R (2019). Novel paradigms in systemic lupus erythematosus. Lancet..

[3] Tektonidou MG, Dasgupta A, Ward MM (2016). Risk of End-stage renal disease in patients with lupus nephritis, 1971-2015: a systematic review and Bayesian meta-analysis. Arthritis Rheumatol.

[4] Ruiz-Irastorza G, Martin-Iglesias D, Soto-Peleteiro A (2020). Update on antimalarials and systemic lupus erythematosus. Curr Opin Rheumatol.

[5] Beck L, Bomback AS, Choi MJ, Holzman LB, Langford C, Mariani LH (2013). KDOQI US commentary on the 2012 KDIGO clinical practice guideline for glomerulonephritis. Am J Kidney Dis.

[6] Kidney Disease: Improving Global Outcomes (KDIGO) Glomerular Diseases Work Group (2021). KDIGO 2021 Clinical Practice Guideline for the Management of Glomerular Diseases. Kidney Int.

[7] Fanouriakis A, Kostopoulou M, Cheema K, Anders HJ, Aringer M, Bajema I (2020). 2019 Update of the Joint European League Against Rheumatism and European Renal Association-European Dialysis and Transplant Association (EULAR/ERA-EDTA) recommendations for the management of lupus nephritis. Ann Rheum Dis.

[8] Schrezenmeier E, Dorner T (2020). Mechanisms of action of hydroxychloroquine and chloroquine: implications for rheumatology. Nat Rev Rheumatol.

[9] Muller-Calleja N, Manukyan D, Canisius A, Strand D, Lackner KJ (2017). Hydroxychloroquine inhibits proinflammatory signalling pathways by targeting endosomal NADPH oxidase. Ann Rheum Dis.

[10] Collins KP, Jackson KM, Gustafson DL (2018). Hydroxychloroquine: a physiologically-based pharmacokinetic model in the context of cancer-related autophagy modulation. J Pharmacol Exp Ther.

[11] Tang C, Livingston MJ, Liu Z, Dong Z (2020). Autophagy in kidney homeostasis and disease. Nat Rev Nephrol.

[12] Liu WJ, Luo MN, Tan J, Chen W, Huang LZ, Yang C (2014). Autophagy activation reduces renal tubular injury induced by urinary proteins. Autophagy..

[13] Liu WJ, Xu BH, Ye L, Liang D, Wu HL, Zheng YY (2015). Urinary proteins induce lysosomal membrane permeabilization and lysosomal dysfunction in renal tubular epithelial cells. Am J Physiol Renal Physiol.

[14] Zhu D, Qu Z, Tan Y, Yu F, Zhao MH (2011). Acute kidney injury in Chinese patients with lupus nephritis: a large cohort study from a single center. Lupus..

[15] Jiang M, Wei Q, Dong G, Komatsu M, Su Y, Dong Z (2012). Autophagy in proximal tubules protects against acute kidney injury. Kidney Int.

[16] Yang C, Xue J, An N, Huang XJ, Wu ZH, Ye L (2018). Accelerated glomerular cell senescence in experimental lupus nephritis. Med Sci Monit.

[17] Pérez de Lema G, Lucio-Cazaña FJ, Molina A, Luckow B, Schmid H, de Wit C (2004). Retinoic acid treatment protects MRL/lpr lupus mice from the development of glomerular disease. Kidney Int.

[18] Tsuruya K, Ninomiya T, Tokumoto M, Hirakawa M, Masutani K, Taniguchi M (2003). Direct involvement of the receptor-mediated apoptotic pathways in cisplatin-induced renal tubular cell death. Kidney Int.

[19] Yang C, Chen XC, Li ZH, Wu HL, Jing KP, Huang XR (2021). SMAD3 promotes autophagy dysregulation by triggering lysosome depletion in tubular epithelial cells in diabetic nephropathy. Autophagy..

[20] McChesney EW (1983). Animal toxicity and pharmacokinetics of hydroxychloroquine sulfate. Am J Med.

[21] Chasset F, Arnaud L, Costedoat-Chalumeau N, Zahr N, Bessis D, Francès C (2016). The effect of increasing the dose of hydroxychloroquine (HCQ) in patients with refractory cutaneous lupus erythematosus (CLE): an open-label prospective pilot study. J Am Acad Dermatol.

[22] Nair AB, Jacob S (2016). A simple practice guide for dose conversion between animals and human. J Basic Clin Pharm.

[23] Reagan-Shaw S, Nihal M, Ahmad N (2008). Dose translation from animal to human studies revisited. FASEB J.

[24] Lee JS, Oh JS, Kim YG, Lee CK, Yoo B, Hong S (2020). Recovery of renal function in patients with lupus nephritis and reduced renal function: the beneficial effect of hydroxychloroquine. Lupus..

[25] Londono Jimenez A, Mowrey WB, Putterman C, Buyon J, Goilav B, Broder A (2018). Brief Report: Tubulointerstitial damage in lupus nephritis: a comparison of the factors associated with tubulointerstitial inflammation and renal scarring. Arthritis Rheumatol.

[26] An N, Chen Y, Wang C, Yang C, Wu ZH, Xue J (2017). Chloroquine autophagic inhibition rebalances Th17/Treg-mediated immunity and ameliorates systemic lupus erythematosus. Cell Physiol Biochem.

[27] Chawla LS, Eggers PW, Star RA, Kimmel PL (2014). Acute kidney injury and chronic kidney disease as interconnected syndromes. N Engl J Med.

[28] Fu Y, Tang C, Cai J, Chen G, Zhang D, Dong Z (2018). Rodent models of AKI-CKD transition. Am J Physiol Renal Physiol.

[29] Yan M, Shu S, Guo C, Tang C, Dong Z (2018). Endoplasmic reticulum stress in ischemic and nephrotoxic acute kidney injury. Ann Med.

[30] Pesce F, Stea ED, Rossini M, Fiorentino M, Piancone F, Infante B (2021). Glomerulonephritis in AKI: from pathogenesis to therapeutic Intervention. Front Med (Lausanne).

[31] Havasi A, Borkan SC (2011). Apoptosis and acute kidney injury. Kidney Int.

[32] He L, Wei Q, Liu J, Yi M, Liu Y, Liu H (2017). AKI on CKD: heightened injury, suppressed repair, and the underlying mechanisms. Kidney Int.

[33] Gao L, Zhong X, Jin J, Li J, Meng XM (2020). Potential targeted therapy and diagnosis based on novel insight into growth factors, receptors, and downstream effectors in acute kidney injury and acute kidney injury-chronic kidney disease progression. Signal Transduct Target Ther.

[34] Moonen L, D’Haese PC, Vervaet BA. Epithelial cell cycle behaviour in the injured kidney. Int J Mol Sci. 2018;19(7). 10.3390/ijms19072038 PMC6073451 30011818

[35] Liu BC, Tang TT, Lv LL, Lan HY (2018). Renal tubule injury: a driving force toward chronic kidney disease. Kidney Int.

[36] Kellum JA, Chawla LS (2016). Cell-cycle arrest and acute kidney injury: the light and the dark sides. Nephrol Dial Transplant.

[37] Yu SM, Bonventre JV (2020). Acute kidney injury and maladaptive tubular repair leading to renal fibrosis. Curr Opin Nephrol Hypertens.

[38] Petri M, Elkhalifa M, Li J, Magder LS, Goldman DW (2020). Hydroxychloroquine blood levels predict hydroxychloroquine retinopathy. Arthritis Rheumatol.

[39] Muthukrishnan P, Roukoz H, Grafton G, Jessurun J, Colvin-Adams M (2011). Hydroxychloroquine-induced cardiomyopathy: a case report. Circ Heart Fail.

[40] Sperati CJ, Rosenberg AZ (2018). Hydroxychloroquine-induced mimic of renal Fabry disease. Kidney Int.

[41] Wang X, Bonventre JV, Parrish AR (2014). The aging kidney: increased susceptibility to nephrotoxicity. Int J Mol Sci.

[42] Schmitt R, Cantley LG (2008). The impact of aging on kidney repair. Am J Physiol Renal Physiol.

[43] Clements ME, Chaber CJ, Ledbetter SR, Zuk A (2013). Increased cellular senescence and vascular rarefaction exacerbate the progression of kidney fibrosis in aged mice following transient ischemic injury. PLoS One.

[44] Zhou B, Wan Y, Chen R, Zhang C, Li X, Meng F (2020). The emerging role of cellular senescence in renal diseases. J Cell Mol Med.

[45] Park JT, Lee YS, Cho KA, Park SC (2018). Adjustment of the lysosomal-mitochondrial axis for control of cellular senescence. Ageing Res Rev.

[46] Tai H, Wang Z, Gong H, Han X, Zhou J, Wang X (2017). Autophagy impairment with lysosomal and mitochondrial dysfunction is an important characteristic of oxidative stress-induced senescence. Autophagy..

[47] Edelstein CL, Venkatachalam MA, Dong Z (2020). Autophagy inhibition by chloroquine and hydroxychloroquine could adversely affect acute kidney injury and other organ injury in critically ill patients with COVID-19. Kidney Int.

[48] Obeidat M, Isaacson AL, Chen SJ, Ivanovic M, Holanda D (2020). Zebra-like bodies in COVID-19: is phospholipidosis evidence of hydroxychloroquine induced acute kidney injury?. Ultrastruct Pathol.

[49] de Almeida DC, Franco MDCP, Dos Santos DRP, Santos MC, Maltoni IS, Mascotte F (2021). Acute kidney injury: incidence, risk factors, and outcomes in severe COVID-19 patients. PLoS One.

[50] Tang TT, Lv LL, Pan MM, Wen Y, Wang B, Li ZL (2018). Hydroxychloroquine attenuates renal ischemia/reperfusion injury by inhibiting cathepsin mediated NLRP3 inflammasome activation. Cell Death Dis.

[51] Wakiya R, Kameda T, Nakashima S, Shimada H, Fahmy Mansour MM, Kato M (2020). Efficacy and safety of hydroxychloroquine therapy for systemic lupus erythematosus patients depend on administration dose. Intern Med.

[52] Wolfe F, Marmor MF (2010). Rates and predictors of hydroxychloroquine retinal toxicity in patients with rheumatoid arthritis and systemic lupus erythematosus. Arthritis Care Res (Hoboken).

[53] Lu J, Huang Y, Ye Q, Shang F, Ming M, Xu H (2021). Low-dose oral hydroxychloroquine led to impaired vision in a child with renal failure: Case report and literature review. Medicine (Baltimore).

[54] Chew CY, Mar A, Nikpour M, Saracino AM (2020). Hydroxychloroquine in dermatology: new perspectives on an old drug. Australas J Dermatol.

[55] Marmor MF, Kellner U, Lai TY, Melles RB, Mieler WF (2016). American Academy of Ophthalmology. Recommendations on Screening for Chloroquine and Hydroxychloroquine Retinopathy (2016 Revision). Ophthalmology..

